# Epidemiology and prognostic factors for new-onset deep venous thrombosis after unicompartmental knee arthroplasty: a retrospective study

**DOI:** 10.1186/s12891-024-07327-y

**Published:** 2024-03-07

**Authors:** Jia Li, Haijing Zhang, Xiaoguang Yu, Guoxing Jia, Sen Liu, Guobin Liu

**Affiliations:** Department of Orthopedic Surgery, The First Hospital of Hebei Medical University, NO. 89 Donggang Road, Shijiazhuang, 050031 Hebei P.R. China

**Keywords:** Unicompartmental knee arthroplasty, Deep venous thrombosis, BMI, Risk factors, D-dimer

## Abstract

**Background:**

Patients who underwent knee joint arthroplasty were at risk of venous thromboembolic events (VTEs), however, less studies were conducted to demonstrate the epidemiology and risk factors of deep venous thrombosis (DVT) following unicompartmental knee arthroplasty (UKA). Objective of this study was to explore the incidence and prognostic factors of DVT after UKA.

**Methods:**

Patients who underwent primary UKA from December 2018 to June 2022 were recruited in this study. Demographic characteristics, operation related variables and laboratory index were extracted and analyzed. Receiver operating characteristic analysis was performed to detect the optimum cut-off value for variables of interest. Univariate and multivariate logistic analysis were performed to identify risk factors of DVT.

**Results:**

351 UKAs with a mean age of 65.4 ± 7.1 years were reviewed. After 12.9 ± 11.2 months follow-up, 35 DVTs were confirmed which indicating an incidence of 9.9%. The results showed that occupation (agricultural laborer) (*P* = 0.008), disease duration > 8.5 years (*P* = 0.035), operation time > 169 min (*P* = 0.003), intraoperative blood loss > 102 ml (*P* < 0.001), BMI > 26.8 kg/m 2 (*P* = 0.001), preoperative D-dimer > 0.29 mg/L (*P* = 0.001), prothrombin time < 10.7 s (*P* = 0.033) and INR < 0.98 (*P* = 0.032) between DVT and Non-DVT group were significantly different. Multivariate logistic regression analysis showed intraoperative blood loss > 102 ml (OR, 3.707; P, 0.001), BMI > 26.8 kg/m 2 (OR, 4.664; P, 0.004) and D-dimer > 0.29 mg/L (OR, 2.882; P, 0.009) were independent risk factors of DVT after UKA.

**Conclusion:**

The incidence of DVT in the present study was 9.9%, extensive intraoperative blood loss, advanced BMI and high level of D-dimer would increase the risk of lower extremity thrombosis by 2–4 times.

## Introduction

Patients who underwent orthopaedic surgery, especially low extremity fractures and joint arthroplasty, were at a higher risk of venous thromboembolic events (VTEs), VTEs includes deep venous thrombosis (DVT), pulmonary embolism (PE), or a DVT progressing to a PE [1]. Main mechanism for the formation of DVT are the hypercoagulable state of blood during the perioperative period and decreased venous blood flow velocity. VTEs of lower limbs would compromise patients’ surgical satisfaction, prolong the length of hospitalization, and increase families’ medical expense, moreover, serious adverse events such as death and pulmonary embolism can be caused by DVT and PE.

According to the published data, 1,100,000 patients received total knee arthroplasty (TKA) in 2012, and the number of populations increased by 11% every year [2]. In China, there were nearly 110 million knee osteoarthritis patients in 2016, and the potential patient population requiring TKA is enormous [3]. Therefore, it is of great clinical and economic significance to explore the epidemiological characteristics and risk factors of VTEs in patients underwent knee arthroplasty.

Compared with TKA, UKA equipped with characteristics of less operation time, lower blood loss and shorten rehabilitation period, some authors pointed out that UKA has a lower incidence of VTE than primary TKA [4, 5]. Based on above condition, we hypothesized that the risk factors of DVT after UKA was different from previous studies. However, less studies were conducted to demonstrate the epidemiology and risk factors of DVT following UKA. Therefore, we designed this retrospective cohort investigation with two purposes: I, to analyze the features of lower extremity DVT after UKA and to track its outcomes; II, to explore the prognostic factors of DVT after UKA, and to assist clinicians in the evaluation and intervention of patients at high risk of VTEs.

## Materials and methods

### Patient

Patients with unilateral or bilateral symptomatic isolated knee osteoarthritis (KOA) treated with UKA at our institution from December 2018 to June 2022 were recruited.

The inclusion criteria were: (i), Patients underwent primary unilateral or bilateral UKA in our joint center; (ii), Preoperative ultrasound of lower extremity venous showed no old thrombosis; (iii), Patients with coagulation system dysfunction (hereditary coagulation disorders, secondary coagulation disorders, increased anticoagulant substances in the circulating blood, or hyperfibrinolysis); (iv), patients routinely underwent VTE assessment and lower extremity venous ultrasonography on the second or third day after surgery.

**Fig. 1:**
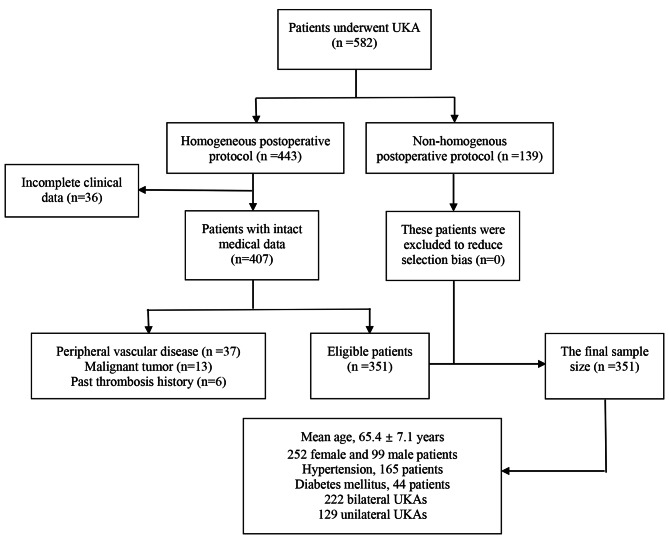
Flow chart of patients underwent UKA and enrolled in this study

The exclusion criteria were: (i), Patients who had taken anticoagulant, antiplatelet and thrombolytic drugs within 1 week before surgery (patients are recommended to stop taking the antiplatelet drugs such as aspirin or clopidogrel for the prevention and treatment of cardiovascular and cerebrovascular diseases before surgery for one week or five days and switch to antibody drugs such as low molecular weight heparin calcium as a substitute); (ii), Patients with a history of peripheral vascular disease of lower extremity, malignant tumor, vascular embolism or a history of infection of the operative limbs; (iii), Patients who received other surgical procedures within 6 months before surgery; (iv), Patients with incomplete clinical data. Flow chart of patients who underwent UKA and enrolled in this study was shown in Fig. 1.

### Surgical technique

All patients enrolled in this study have undergone cemented mobile-bearing UKA. Once the tibial saw guide assembled in situ, the vertical and horizontal tibial cut is performed, and milling the condyle when the microplasty femoral drill guide is centrally positioned. Second and third milling is necessary to obtain an ideal balance during flexion and extension of knee joint. The posterior medial edge of the tibial template is aligned with the posterior medial cortex of the tibia to obtain a suitable tibial prosthesis model, then insert the twin peg femoral trial component and meniscal bearing of the chosen thickness.

### Postoperative protocol for DVT

Ice therapy, the knee-flexed position and elastic bandage were introduced as measures to relieve wound pain and swelling postoperatively. 2500AXa of low-molecular-weight heparin will be given subcutaneously 12 h after UKA, meanwhile 5000AXa or 10000Axa of low-molecular-weight heparin was applied to patients routinely for prevention and treatment of venous thrombosis of lower extremity since the day after surgery. Ankle pump movements and active or passive knee flexion exercise were encouraged immediately after surgery, patients are encouraged to undergo partial and full weight-bearing walking activities with the assistance of walking aid 6 h or the first day after surgery. Generally, UKA patients will be discharged form hospital 3–4 days after operation, and patients were recommended to continue take oral rivaroxaban of 10 mg/day for the prevention of DVT.

### Data collection

Three aspects of clinical data were extracted from electronic medical record and face-to-face interviews will be conducted if necessary to collect variables that may affect the occurrence of DVT following UKA. Demographic characteristic includes age, gender, occupation, place of residence, disease duration (previous knee osteoarthritis history), body mass index (BMI), hypertension, diabetes, and other comorbidities. Surgical related variables: operation time, length of incision, interoperative blood loss, tourniquet working time, perioperative dose of tranexamic acid and so on. Laboratory test index: erythrocyte count (RBC, 10^9^/L), hemoglobin (HGB, g/L), platelet count (PLT, 10^9^/L), erythrocyte sedimentation rate (ESR, mm/h), prothrombin time (s), international normalized ratio (INR), D-dimer (mg/L), C-reactive protein (CRP, mg/L), serum albumin (ALB, g/L), total protein (TP, g/L), seroglobulin (GLB, g/L) and etc.

### Diagnosis of VTE

Ultrasonographic scans were performed by a senior professional physician, B-mode ultrasonography with compression and color Doppler imaging were performed to evaluate the bilateral common femoral veins, superficial veins, popliteal veins, and calf veins using an Epiq7 (Philips, Netherlands) sonographic scanner with a linear transducer. The reference standards for diagnosis of venous thrombosis are: (1) the venous lumen cannot be compressed or only partially closed; (2) color Doppler shows an incompletely filled blood flow of lumen.

This study was approved by the Institutional Review Board of our hospital, and informed consents from all the study participants were collected.

### Statistical analysis

Continuous variables were expressed as the mean ± SD, whitney U-test was used for non-normally distributed continuous variables, t-test for normally distributed variables and the Chi square test for categorical data. Univariate and multivariate logistic regression analysis was performed to identify independent risk factors of DVT. The Hosmer–Lemeshow test was used to evaluate goodness-of-fit of the final model, and an acceptable fitness was enacted as *P* > 0.05. Receiver operating characteristic (ROC) analysis was performed to detect the optimum cut-off value for continuous variables. Values of *P* < 0.05 were considered to indicate a significant difference. Statistical procedures were performed by SPSS 19.0 software package (SPSS Inc., Chicago, Illinois).

## Results

Finally, a total of 351 patients underwent homogenized perioperative management following UKA with a mean age of 65.4 ± 7.1 years (ranges from 49 to 87 years) were retrospectively reviewed. There were 99 male (28.2%) and 252 female (71.8%) patients in this study, 222 and 129 patients underwent bilateral and unilateral UKA (Fig, 1), and mean previous knee arthritis history for these patients was 7.1 ± 6.0 years. In terms of comorbidities, 165 and 44 patients have hypertension or diabetes mellitus respectively. The operation time ranges from 75 to 210 min (131 ± 42.9 min), length of surgical incision range from 8 to 17 cm (11.9 ± 0.9 cm) and mean hospitalization was 12.9 ± 4.6 days for all patients.

**Fig. 2:**
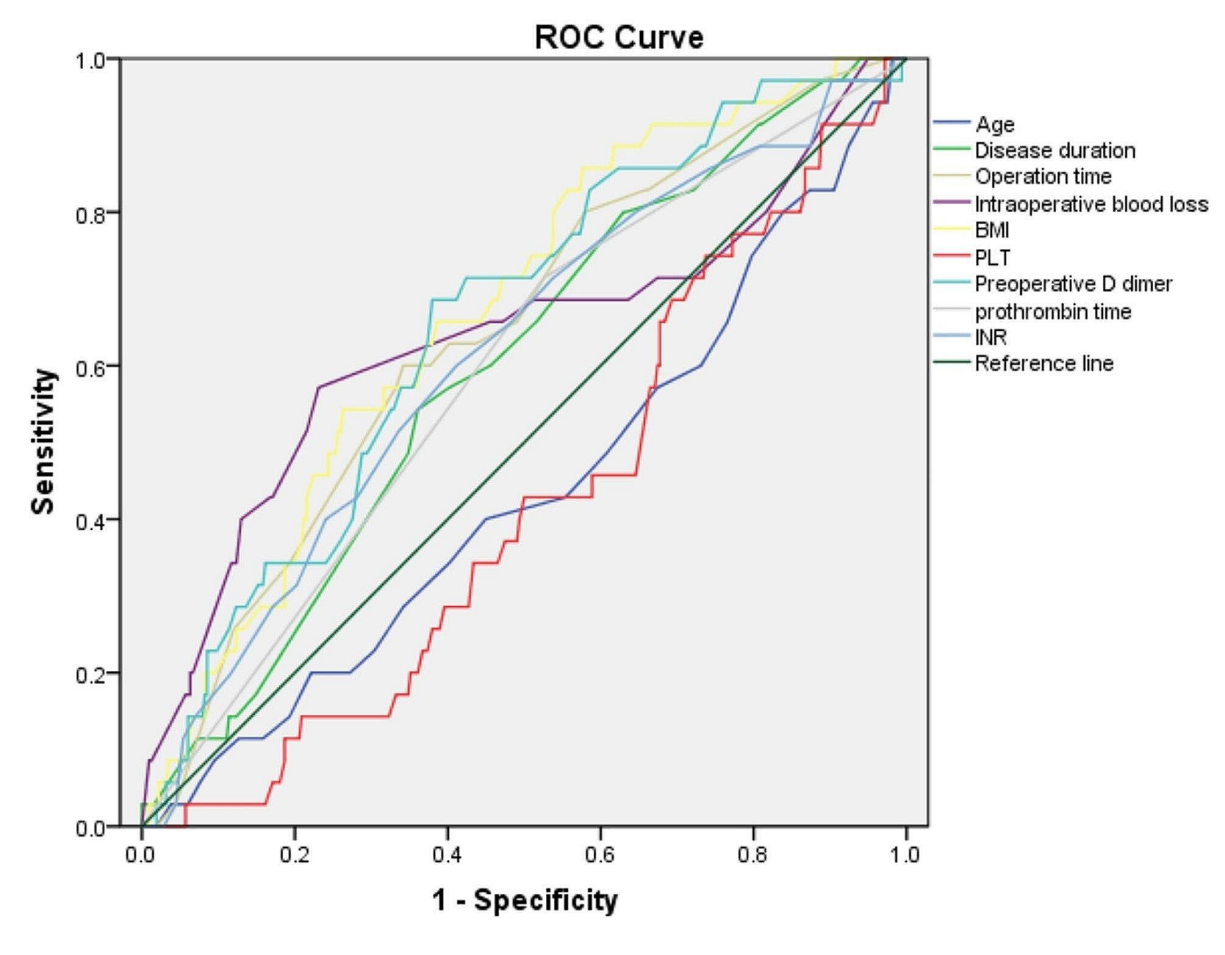
Receiver operating characteristic (ROC) analysis of some variables of interest

Figure 2; Table 1 summarized the optimal cut-off value, area under the curve (AUC) and its corresponding 95% CI for some interested continuous variables. It was defined that variables with statistical significance when the AUC of ROC analysis was greater than 0.5. According to the P value, disease duration > 8.5 years, operation time > 169 min, intraoperative blood loss > 102 ml, BMI > 26.7 kg/m^2^, preoperative D-dimer > 0.29 mg/L, prothrombin time < 10.7s and INR < 0.98 were finally analyzed in the univariate analysis and multivariate logistic regression model to demonstrate the risk factors of DVT.

**Table 1:** Optimal cut-off value and area under the curve (AUC) of variables in ROC analysis

Variables	Cut-off value	AUC	95%CI	*P* value
Age (year)	51.5	0.437	0.335–0.539	0.223
Disease duration (year)	8.5	0.598	0.508–0.690	0.057
Operation duration (min)	169	0.642	0.550–0.734	0.006‡
Intraoperative blood loss (ml) BMI (kg/m^2^)	102 26.7	0.636 0.670	0.522–0.750 0.583–0.756	0.008‡ 0.001‡
Preoperative- PLT (10^9^/L)	134	0.420	0.329–0.510	0.120
D dimer (mg/L)	0.29	0.655	0.566–0.745	0.003‡
Prothrombin time (s)	10.7	0.622	0.524–0.720	0.018‡
INR	0.98	0.614	0.516–0.711	0.027‡

‡, *P* < 0.05; BMI, body mass index; PLT, platelet count; INR, international normalized ratio

After a mean 12.9 ± 11.2 months follow-up period, 35 DVTs were confirmed which indicating an incidence rate of 9.9%. There were 2 cases of tibiofibular vein thrombosis, one posterior tibial veins thrombosis and 32 cases of muscular calf vein thrombosis in the 35 DVTs. Table 2 presented the difference between patients with and without DVT. The results showed occupation (agricultural laborer) (*P* = 0.008), disease duration > 8.5 years (*P* = 0.035), operation time > 169 min (*P* = 0.003), intraoperative blood loss > 102 ml (*P* < 0.001), BMI > 26.8 kg/m^2^ (*P* = 0.001), preoperative D-dimer > 0.29 mg/L (*P* = 0.001), prothrombin time < 10.7 s (*P* = 0.033) and INR < 0.98 (*P* = 0.032) between DVT and Non-DVT group were significantly different. Subsequently, all variables mentioned above were transferred into a binary logistic regression analysis model, finally, intraoperative blood loss > 102 ml (OR, 3.707; P, 0.001), BMI > 26.8 kg/m^2^ (OR, 4.664; P, 0.004) and D-dimer > 0.29 mg/L (OR, 2.882; P, 0.009) were identified as independent risk factors of postoperative DVT in patients underwent primary UKA (Table, 3). The P value of Hosmer–Lemeshow test of the first and final step were 0.206 and 0.177 respectively, which demonstrated a goodness-of-fit for the multivariate logistic regression analysis model.

**Table 2:** Univariate analysis of factors associated with postoperative deep venous thrombosis (DVT)

Variables	DVT Group (*n* = 35, %)	Non-DVT Group (*n* = 316, %)	*P* value
Age > 51.5 year Gender Male Female Occupation Agricultural laborer Office clerk Residence, rural area	35 (100.0) 6 (17.1) 29 (82.9) 21 (60.0) 14 (40.0) 16 (45.7)	310 (98.1) 93 (29.4) 223 (70.6) 252 (79.7) 64 (20.3) 176 (55.7)	0.411 0.125 0.008‡ 0.260
Disease duration > 8.5 year Comorbidities Hypertension Diabetes	19 (54.3) 21 (60.0) 4 (11.4)	114 (36.1) 144 (45.6) 40 (12.7)	0.035‡ 0.105 0.835
Length of surgical incision (cm) Operation time > 169 min Intraoperative blood loss > 102 ml BMI > 26.8 kg/m^2^	11.8 ± 0.5 21 (60.0) 20 (57.1) 30 (85.7)	11.9 ± 1.0 108 (34.2) 73 (23.1) 182 (57.6)	0.410 0.003‡ < 0.001‡ 0.001‡
Preoperative- WBC (3.5–9.5*10^9^/L)	5.6 ± 1.5	5.9 ± 1.4	0.359
RBC (3.8–5.1*10^9^/L) HGB (115–150* g/L) PLT (125–350*10^9^/L) D dimer > 0.29 mg/L Prothrombin time < 10.7 s INR < 0.98 Length of hospitalization (day)	4.3 ± 0.4 131.4 ± 14.2 221.8 ± 44.7 24 (68.6) 10 (28.6) 14 (40.0) 11.7 ± 3.9	4.3 ± 0.4 132.8 ± 15.0 239.2 ± 64.4 121 (38.3) 150 (47.5) 186 (58.9) 13.1 ± 4.6	0.446 0.594 0.121 0.001‡ 0.033‡ 0.032‡ 0.087

‡, significant variable; WBC, white blood cell count; RBC, erythrocyte count; HGB, hemoglobin

**Table 3:** Multivariate logistic analysis of risk factors associated with postoperative DVT

Variables	OR	95%CI	*P* value
Intraoperative blood loss > 102 ml	3.707	1.711–8.029	0.001
BMI > 26.8 kg/m^2^	4.664	1.647–13.209	0.004
D dimer > 0.29 mg/L	2.882	1.299–6.393	0.009

## Discussion

Venous thrombosis was reported as a common complication after orthopaedic surgery, especially for trauma patients of low extremity fractures. UKA is a cost-effective surgical treatment option in relieving the pain of knee joint, however, although UKA has more surgical and rehabilitative advantages than TKA, patients underwent UKA were also particularly prone to develop DVT. An increased number of elderly degenerative knee arthritis patients along with the popularity of UKA, it is of great clinical significance to explore the characteristics and prognosis of DVT after UKA. Therefore, we designed this observational study to demonstrate the epidemiological features and risk factors of DVT following UKA. The findings of our investigation have positive significance in helping clinicians to screen out patients with high risk of postoperative thrombosis and formulate corresponding perioperative medical interventions.

Here, incidence of DVT after UKA was 9.9% in the present study, this data was consistent with previous published reports. Incidence of DVT was reported low in knee arthroscopy, Krych et al. [1] retrospectively reviewed 12,595 consecutive knee arthroscopies, they found 0.3% patients were confirmed DVT and 0.06% for PE. By contrast, the rate of DVT in patients underwent osteotomies around the knee was exceedingly high, postoperative DVT was detected in 11.9% knees after open wedge high tibial osteotomy and 22.6% in later closed wedge high tibial osteotomy respectively [6]. As for VTEs after joint arthroplasty surgery, incidence of DVT ranges from 0.4 to 42% [7, 8], occurrence of DVT of lower extremity following knee arthroplasty, especially the UKA surgery, is lower than that of hip arthroplasty. In our clinical practice, patients were encouraged to perform ankle pump exercise on the day after operation, meanwhile, pneumatic therapy was also introduced to promote lower limb blood circulation. More importantly, anticoagulants such as low-molecular-weight heparin was routinely injected subcutaneously in postoperative patients. Overall, the above comprehensive measures together can reduce the risk of DVT in patients underwent UKA procedure.

BMI, a variable which widely concerned by clinicians and researchers, has been reported to be associated with various postoperative complications in the published studies. In elective open ventral hernia repair surgery, it was also confirmed that increased BMI is often accompanied by a stepwise increase in surgical site infection (SSI) rate [9]. Even for pediatric colorectal patients, incremental increase in BMI by 1.0 kg/m^2^ was associated with 4.3% increased likelihood of developing deep incisional SSI and 2.3% increase of superficial wound disruption [10]. Moreover, morbid obesity was associated with an increased risk of acute kidney injury, urinary tract infection, readmission, and overall minor complications when compared to normal BMI patients following ankle arthrodesis [11]. However, some authors advocated that perioperative surgical complications were not related to BMI but co-existing comorbidities following obesity [12]. In the present study, we have confirmed the relationship between DVT and BMI, and risk of DVT would upgrading by 4-folds when BMI > 26.8 kg/m^2^. Comorbidities such as hypertension, diabetes which may be caused by advance BMI were also analyzed, however, the results showed that these comorbidities did not significantly increase the incidence of thrombotic events. According to the Chinese reference criteria, BMI was divided into six groups, namely: <18.5, underweight; 18.5–23.9, normal; 24-27.9, overweight; 28-31.9, obesity; ≥32, morbid obesity. There were 9, 15 and 9 DVTs in overweight (9/128), obesity (15/120) and morbid obesity (9/48) patients with an occurrence rata of 7.0%, 12.5% and 18.8% respectively. Antithrombin (AT) is the most important anticoagulant in plasma and tissue plasminogen activator (t-PA) and tissue plasminogen activator inhibitor (PAI) is also paly an important role in the fibrinolytic system. The activity of AT is significantly decreased when patients are accompanied by obesity and other risk factors [13–15]. The possible mechanism of higher BMI would increase incidence of DVT is that the level of PAI would increase and activity of t-PA decrease significantly in overweight and obesity patients, which suggesting that fibrinolytic function may compromise with the advanced BMI.

D-dimer, a specific production of fibrin degradation which concentration can vary with factors such as trauma, surgery, pregnancy and thrombosis, mainly reflects the function of body’s anticoagulation fibrinolytic system. Serum D-dimer test is introduced to assist clinical auxiliary diagnosis of thrombotic disease, and has significant role in dynamically evaluating the risk of DVT, this index has the characteristics of rapidity and sensitivity making it an important measurement for the prevention of VTEs. According to the results of our research, when the level of serum D-dimer exceeds 0.29 mg/L, risk of postoperative DVT will increase by 2.8 times. Jiang et al. [16] have analyzed the risk factors of VTEs in patients underwent knee arthroplasty, they found D-dimer > 0.5 ug/ml was positively correlated with DVT (OR, 4.441; 95% 1.942–10.153). Similar results were also reported in arthroscopic posterior cruciate ligament reconstruction surgery [17], which concluded an increased post-surgery D-dimer value was significantly associated with an elevated risk of DVT. However, both of those studies did not further explore the precise value of D-dimer which could more accurately predict the occurrence of DVT. In our study, the optimal cut-off value of preoperative D-dimer was determined by ROC analysis, namely 0.29 mg/L, and the strong correlation between this value and DVT was also demonstrated by univariate and multivariate regression analysis models. This finding plays an important role in assisting clinicians to actively and accurately estimate patients’ postoperative risk of lower limb thrombosis, meanwhile, formulate and modify the relevant treatment strategy. We believe that the in-depth analysis of D-dimer is one of the important innovations of our investigation when compared with previous studies.

We found that occurrence of DVT was also positively correlated with an excessive blood loss. In our study, 63.3% patients underwent bilateral UKAs, therefore the mean intraoperative blood loss for the included sample was 96.1 ± 47.2 ml. ROC and multivariate logistic regression analysis showed blood loss during surgery more than 102 ml was independent risk factors of DVT. Surgical stress often leads to hemodynamic instability and affects blood circulation, which can contribute to the formation of VTEs. Relationship between operation time and postoperative complications in orthopaedic surgery and other disciplines were demonstrated in many articles [18–20]. UKA only replaces the severely degenerated medial compartment, therefore, the process of osteotomy and gap balancing is simple compared to TKA, and a shorter operative time and blood loss can be achieved. In addition, with the use of tourniquets, UKA always accompanied by less blood loss, moreover the bleeding is mostly concentrated in the subcutaneous area, infrapatellar fat pad, and other relatively constant positions, which making physical hemostasis easier. The joint capsule and subcutaneous tissue were injected with a “cocktail” injection during surgery, these can also help reduce intraoperative bleeding, finally these elements together may lead low cut-off value for intraoperative blood loss to an independent risk factor of postoperative DVT. However, there is no doubt that operative duration of bilateral UKA will significantly increase. For patients who received TKA even using standard therapy of low molecular weight heparin for prophylactic treatment of DVT, bilateral surgery would also increase the risk of VTEs by 4-folds [21]. Therefore, physicians should make recommendations of diagnosis and treatment to patients on the premise of careful physical examination and accurate evaluation of imaging data, and simultaneous bilateral arthroplasty should be replaced by staged steps if possible. In clinical practices, in order to maintain the hemodynamic stability of patients, preoperative infusion of crystalloid and colloidal solution can effectively supplement blood volume and improve the body’s tolerance to hypovolemia, and this intervention may inherit great significance in preventing and reducing the occurrence of perioperative DVT.

It should be pointed out that our study is not without limitations. First of all, the retrospective study design inevitably possesses selection and confounding bias; 351 UKAs were reviewed in this investigation and the sample size of this study was relatively small, however, as a single-center study the capacity was acceptable. Secondly, variables such as frequency and duration of ankle pump exercise were not extracted and analyzed, which may have some important influence on the occurrence of DVT. Finally, an ideal algorithm should be proposed to calculate the risk of developing DVT based on the combination of the three significant factors founded in this study. Despite these limitations, reliability of our study was strengthened by multiple statistical models and we have demonstrated the precise value of some prognostic risk factors.

## Conclusion

In summary, this study explored the epidemiologic features of postoperative DVT and demonstrated independent risk factors in patients underwent primary UKA. Incidence of DVT in the present study was 9.9%, what’s more, extensive intraoperative blood loss, advanced BMI and high level of D-dimer would increase the risk of lower extremity thrombosis by 2–4 times.

## Data Availability

The datasets generated and/or analysed during the current study are not publicly available due to the sake of patient privacy but are available from the corresponding author on reasonable request.

## Abbreviations

VTEs: venous thromboembolic events
DVT: deep venous thrombosis
TKA: total knee arthroplasty
UKA: unicompartmental knee arthroplasty
KOA: knee osteoarthritis
BMI: body mass index
RBC: erythrocyte count
INR: international normalized ratio
CRP: C-reactive protein
ALB: serum albumin
TP: total protein
GLB: seroglobulin
ROC: receiver operating characteristic
AUC: area under the curve
